# Contrast Negation and Texture Synthesis Differentially Disrupt Natural Texture Appearance

**DOI:** 10.3389/fpsyg.2012.00515

**Published:** 2012-11-20

**Authors:** Benjamin Balas

**Affiliations:** ^1^Department of Psychology, Center for Visual and Cognitive Neuroscience, North Dakota State UniversityFargo, ND, USA

**Keywords:** texture discrimination, texture synthesis, image statistics

## Abstract

Natural textures have characteristic image statistics that make them discriminable from unnatural textures. For example, both contrast negation and texture synthesis alter the appearance of natural textures even though each manipulation preserves some features while disrupting others. Here, we examined the extent to which contrast negation and texture synthesis each introduce or remove critical perceptual features for discriminating unnatural textures from natural textures. We find that both manipulations remove information that observers use for distinguishing natural textures from transformed versions of the same patterns, but do so in different ways. Texture synthesis removes information that is relevant for discrimination in both abstract patterns and ecologically valid textures, and we also observe a category-dependent asymmetry for identifying an “oddball” real texture among synthetic distractors. Contrast negation exhibits no such asymmetry, and also does not impact discrimination performance in abstract patterns. We discuss our results in the context of the visual system’s tuning to ecologically relevant patterns and other results describing sensitivity to higher-order statistics in texture patterns.

## Introduction

Texture perception supports a wide range of visual tasks (Landy, under review). Natural texture appearance can be used to estimate material properties (Motoyoshi et al., 2007), infer 3D shape (Li and Zaidi, 2000; Fleming et al., 2004, 2011), and segment boundaries in complex scenes (Malik and Perona, 1990; Malik et al., 2001). Texture processing, by which we refer to whatever it is the visual system does to recognize “stuff” rather than “things,” (Adelson and Bergen, 1991) is usually conceived of implicitly or explicitly in terms of a summary of visual information (e.g., feature histograms). Determining what measurements the visual system uses to encode texture appearance remains an open and important question. Besides the visual tasks described above, understanding the code for texture appearance may also be relevant for characterizing the constraints on visual performance in a far wider range of tasks. Recent results suggest that the nature of texture representations (and their limitations) may explain performance in crowding tasks (Balas et al., 2009), visual search (Rosenholtz et al., 2012b); and provide a language for describing the effects of visual attention (Rosenholtz et al., 2012a). Texture representations are lossy – typically, information about where specific features were in the image is lost, but aggregate data about the frequency and co-occurrences of image features is retained. The transformation implemented by processing an image via texture representations may be the basis of a distinct mode of processing that governs much of what see in the visual periphery (Freeman and Simoncelli, 2011; Rosenholtz, 2011) leading to a wide range of effects that follow from “seeing sidelong” (Lettvin, 1976).

We have referred to texture processing as a lossy image transformation since there are many circumstances in which micropatterns that are easily distinguishable in isolation nonetheless are indistinguishable in the context of a texture field (Julesz, 1981; Beck, 1983). Clearly some information is unavailable in a texture representation, but what information is included and what is left out? There are multiple image statistics that the human visual system is known to be sensitive to. If we just consider summary statistics that can be applied to the intensity histogram of independent and identically distributed (IID) textures, for example, observers’ discrimination ability relies upon three mechanisms: the mean, variance, and “blackshot” (sensitivity to the darkest elements) of candidate textures (Chubb et al., 1994, 2004). Observers are also sensitive to the skewness of the intensity histogram of natural textures, which alters perceived glossiness (Motoyoshi et al., 2007; Sharan et al., 2008). The power spectrum of natural textures contains information that can be used to predict shape from texture (Li and Zaidi, 2001) and observers’ judgments of texture similarity (Balas, 2008). The power spectrum’s magnitude roll-off factor (the coefficient β, for images characterized by a 1/f^β^ spectrum) is also a useful predictor of roughness judgments for fractal surfaces (Padilla et al., 2008). Finally, the visual system is also known to be sensitive to higher-order statistics in natural textures. Higher-order pixel statistics (relationships between “needles,” triangles, and higher-order structures created by jointly measuring n-tuples of intensities) can be balanced to extreme degrees (Julesz et al., 1978; Tyler, 2004), yet lead to visually discriminable textures. The discriminability of natural textures from synthetic textures created by matching wavelet coefficients at multiple scales (Heeger and Bergen, 1995) also demonstrates sensitivity to higher-order statistics – if observers only used histograms of oriented edge filter outputs to encode texture appearance, then synthetic textures created by the Heeger–Bergen algorithm should be indistinguishable from the real thing. Similarly, synthetic textures that use joint encoding of wavelet coefficients (Portilla and Simoncelli, 2000) are also discriminable from their parent images in the visual periphery (Balas, 2006) depending on the subset of joint statistics that are permitted to contribute to the representation of appearance. Were the Portilla–Simoncelli model a complete model of texture perception, natural textures should be indistinguishable from the synthetic textures created by the algorithm – the fact that they are discriminable indicates that participants use information beyond what is in the model. Texture perception thus relies on a number of summary statistics that encode visual appearance, some of which have been identified and are known to contribute to specific tasks and specific aspects of appearance, and some of which remain unspecified but nonetheless reveal themselves by their absence from synthetic textures that are imperfect copies of natural images.

In the current study, we examined what information observers use to discriminate natural textures from unnatural ones. Specifically, we defined two kinds of unnatural texture corresponding to two transformations of natural texture appearance: contrast negation and parametric texture synthesis. These transformations are of particular interest for several reasons. First, each transformation preserves a well-defined set of features. Contrast negation preserves orientation energy and the *isophotes* in a texture image (Fleming and Bulthoff, 2005), while texture synthesis preserves the set of image statistics that form the basis of the model under consideration. Second, each transformation also disrupts specific aspects of appearance. Contrast negation reverses edge polarity (a 180° change in phase), while summary statistics beyond the scope of a particular texture synthesis model are not likely to be constrained in a synthetic image. For example, the Heeger–Bergen model does not measure the co-occurrence of filter outputs – in this context, joint relationships between filters are considered beyond the scope of that particular model and are in practice not matched between original and synthetic textures. We assume that both of these transformations cause images of natural textures to deviate from the properties to which the human visual system is tuned (Tkacik et al., 2010).

We conducted two experiments using a 4AFC detection paradigm that required observers to identify a target oddball texture patch from an array of unique texture patches. Since no two images in the array were identical, participants had to perform this task by comparing texture appearance across patches rather than relying on simpler image-level strategies (e.g., pixel matching). In each task, we asked how easy it was for observers to discriminate natural textures from unnatural textures subject to some transformation applied to the entire array of textures. In Experiment 1, we asked how the ability to distinguish between natural and synthesized textures was affected by the positive or negative contrast polarity of the entire array. In Experiment 2, we asked how the ability to distinguish between natural and contrast-negated textures was affected by replacing the original patches comprising an array with synthetic versions of the same. In each case, the crucial question was whether the transformation applied to the entire array (contrast negation in Experiment 1, texture synthesis in Experiment 2) affected the discrimination task under consideration – if so, then observers must have been using information in the original images that was no longer available in the transformed images.

In both tasks, we also examined whether detection of the oddball texture was affected by the appearance of the oddball. That is, is there an asymmetry for detecting a natural texture among unnatural distractors, or vice versa? Balas (2006) used both types of oddball to discourage observers from reaching high levels of performance via trivial strategies, but did not analyze error rates separately for these conditions. Here we do so, since the presence or absence of such an asymmetry tells us whether or not the transformation applied to the unnatural textures in each task homogenizes distractor appearance. If, for example, patches from a synthetic texture are more homogenous than patches from its original parent texture, an oddball from the original image should be easier to detect than an oddball from the synthetic image by virtue of the decreased variability in distractor appearance (Rosenholtz, 2000, 2001). Finally, we compared performance with familiar, real-world textures (fruit and vegetable textures) to performance with unfamiliar, abstract artwork. Comparing the results across these two categories served an important purpose. This comparison allowed us to assess the contribution of artifacts arising purely from the method of transforming our natural textures into their unnatural counterparts, ruling out effects driven solely by idiosyncrasies of the synthesis or negation processes. The abstract artwork textures that we used lacked discernible objects, were clearly planar, and lacked a wide range of interesting material properties. In short, though they contained extended contours, differently oriented structures, and a range of pixel intensities, they lacked many other qualities of textures we typically encounter. Should any effects we observe depend solely on image artifacts introduced during contrast negation or texture synthesis, there should be no interaction with stimulus category – the same artifacts should be evident in both types of texture. An interaction with texture category, on the other hand, would tell us that there is a crucial difference between removing information from a natural image and removing information from a generic complex pattern.

## Experiment 1

In our first experiment, we examined how observers’ ability to distinguish natural and synthetic textures was affected by the contrast polarity of the images under consideration. We used a 4AFC oddball detection paradigm to measure discrimination performance for two texture classes: (1) fruits and vegetables, and (2) Abstract artwork. We included Abstract paintings as a control condition since these images share the general 1/f profile of spatial frequency that natural scenes and textures exhibit (Taylor et al., 2011), but lack most other properties of real-world textures (e.g., shape-from-shading, material properties, segmentable objects).

### Methods

#### Subjects

We recruited 13 participants (eight female) to take part in Experiment 1. Participants were between the ages of 18–22 years old and self-reported normal or corrected-to-normal vision.

#### Stimuli

**Figure 1:**
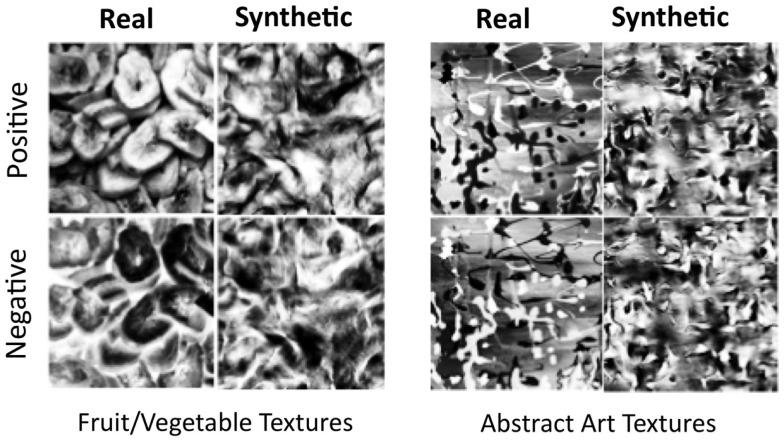
**Examples of textures from the fruit/vegetable category (left) and the abstract art category (right)**. Original textures in each category were transformed into synthetic textures using the Portilla–Simoncelli algorithm (right column of each panel) and were also contrast-negated (bottom row of each panel).

We selected 16 grayscale textures for use in this task. Eight of these textures depicted fruits and vegetables and the remainder depicted abstract artworks (Figure 1). We chose textures in both categories to be fairly homogeneous, to be well-matched according to the scale of the constituent items in the texture, and to have fairly flat intensity distributions. All images were 256 × 256 pixels in size and contained 256 gray-levels. The power spectrum magnitude roll-off of these textures (1/f^β^) did not differ by category [Average β_FruitVegetable_ = −2.36, Average β_AbstractArt_ = −2.20, *t*(18) = −1.01, *p* = 0.33, two-tailed independent-samples *t*-test].

We used each original texture to create three transformed textures: (1) A contrast-negated version of the original texture, (2) a synthetic version of the original, and (3) a contrast-negated synthetic version of the original – this was created by contrast-negating the same synthetic texture described in (2). Contrast negation was carried out using the GIMP image-editing application. Parametric texture synthesis was performed in Matlab using the Portilla–Simoncelli algorithm (Portilla and Simoncelli, 2000), which matches synthetic texture appearance to an original texture based on a texture code that is largely comprised of correlations between complex wavelet coefficients. We chose this algorithm since it has recently been used in a range of studies characterizing peripheral vision (Balas, 2006; Balas et al., 2009; Rosenholtz et al., 2012b). A detailed description of the algorithm can be found in the original manuscript, or in Balas et al. (2009). There are, of course, several alternative techniques for synthesizing natural textures. The most successful methods for graphics applications, however, are non-parametric methods that use image quilting or similar approaches to *reproduce* textures without *representing* them in terms of a consistent vocabulary of image features (Efros and Leung, 1999; Efros and Freeman, 2001). As such, the relationship between these algorithms and the human visual system is more difficult to interpret, and so we have opted to use a robust parametric model here.

Synthetic textures were generated based on the default parameters of the Portilla–Simoncelli model and 50 iterations of the synthesis procedure. We obtained good convergence for all of the syntheses, as measured by near-zero changes in average pixel intensity at the final iteration (indicating a local minimum for matching image statistics between the parent and synthetic image) and also by close agreement between the texture statistics of the original image and the synthetic image (indicating that the local minimum attained is globally good). Adjusting the parameters of the P-S model will certainly have an impact on the outcome of the synthesis procedure, and we opted to use the default parameters here because we were unable to identify a set of parameter values that yielded synthetic textures that were of subjectively higher quality. In principle, increasing the number of orientations, or changing the spatial neighborhood over which correlations are stored could change synthetic texture appearance to more closely match target images, but in practice, we found that the default parameters were difficult to consistently improve upon over the set of images we chose to test here.

Finally, all images were divided into quadrants, and each quadrant was further cropped with a circular window 128 pixels in diameter. Each image thus yielded four non-overlapping circular patches. The intensity (pixel values) histogram of each patch was equalized, so that simple pixel statistics (e.g., mean luminance) could not be used to discriminate one patch from another.

#### Procedure

**Figure 2:**
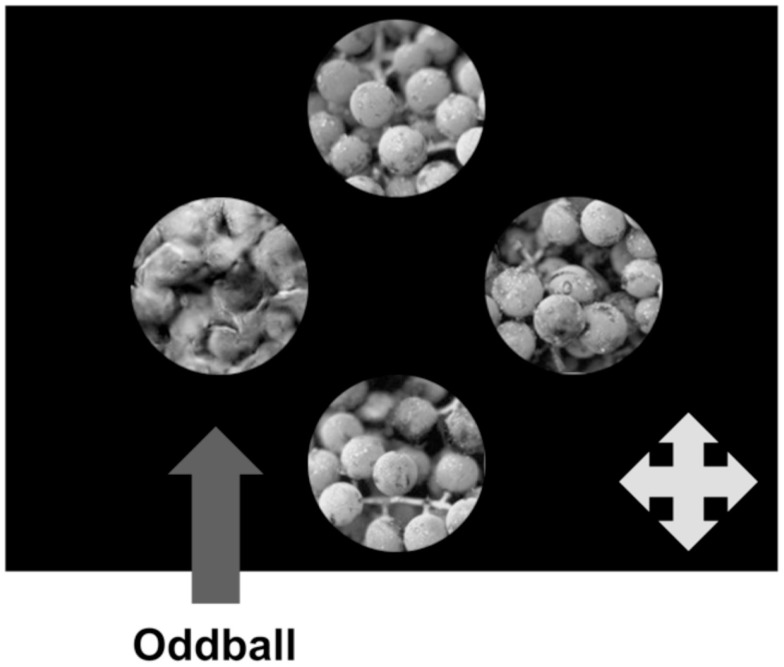
**Schematic representation of a single trial in Experiment 1**. The four texture patches are unique, and in this instance the target oddball patch is the single synthetic patch in an array of real patches. Participants’ task was to identify the location of the oddball (Top, Bottom, Left, or Right) as quickly and accurately as possible.

We asked participants to complete a 4AFC oddball detection task using the original and transformed stimuli described above. In this experiment, target texture patches differed from distractors by virtue of real/synthetic appearance. On each trial, participants simultaneously viewed four unique texture patches: three non-overlapping patches from one larger image, as described above, and another which was the target “oddball” stimulus. The texture patches were presented on a uniform black background, arranged in a cross-shape (Figure 2) with the location of the oddball texture randomized on each trial. Participants identified the position of the oddball on each trial using a USB gamepad.

Participants were free to view each array of texture patches for an unlimited amount of time, but were encouraged to correctly identify the oddball as quickly as possible. Eye movements were not constrained, so participants were free to foveate all of the texture patches on each trial. Since eye movements were not proscribed, observers who made multiple fixations across the extent of our stimulus array accumulated information from both foveal and peripheral vision. Critically, since all of the image patches presented on a single trial were unique, an observer cannot succeed at this task by attempting to scrutinize and match individual micropatterns, even though presentation is unlimited.

We presented the stimuli on a 1024 × 768 display that was positioned at a distance of 60 cm from the participant. This distance was controlled using a Headspot chin rest, which was adjusted so that the display was situated at eye-level. At this viewing distance, each patch subtended ∼2° of visual angle.

We manipulated several properties of the stimulus array in Experiment 1. First, the entire array of textures could be comprised either of contrast-positive or contrast-negative versions of the patches. This manipulation allowed us to examine the extent to which discriminating between real and synthetic textures depended upon visual features that are specific to natural contrast polarity. Second, we varied the appearance of the oddball across trials such that half of the time the oddball patch was the only real texture presented among synthetic distractors, and half of the time the opposite was the case. By varying the nature of the oddball, we were able to assess whether or not there was an extant asymmetry in identifying a real vs. synthetic oddball, which is an important indicator that distractor appearance may be more homogenous in one case than the other (Rosenholtz, 2001). Specifically, we hypothesized that texture synthesis may homogenize the appearance of distinct patches due to the loss of higher-order statistics, which would lead to an advantage for detecting real oddballs among synthetic distractors. Finally, we asked observers to perform this task using both our set of natural object textures (fruits and vegetables) and our set of abstract art textures. These texture categories were presented to participants in separate blocks, with block order alternated across participants and oddball type and contrast polarity randomized within each block. By comparing the effects of oddball type and contrast polarity across categories, we were able to determine whether or not the impact of negation and texture synthesis was specific to categories observers have substantial experience with.

Participants completed 48 trials per condition, for a grand total of 384 trials (192 per block). Participants typically completed the entire experiment in ∼25 min. All stimulus display and response collection routines were implemented in Matlab using the Psychtoolbox extensions (Brainard, 1997; Pelli, 1997). All experimental procedures and recruitment methods were approved by the North Dakota State University IRB.

#### Results

We measured participants’ accuracy and response latency to correct answers in all conditions. We found that response latencies tended to be quite long (∼2.5 s per trial averaged across all participants) and were also highly variable within individual subject’s data. As such, we chose not to analyze these data further in this task or Experiment 2, since it was not clear how best to summarize the data from individual participants in terms of standard measures of central tendency.

**Figure 3:**
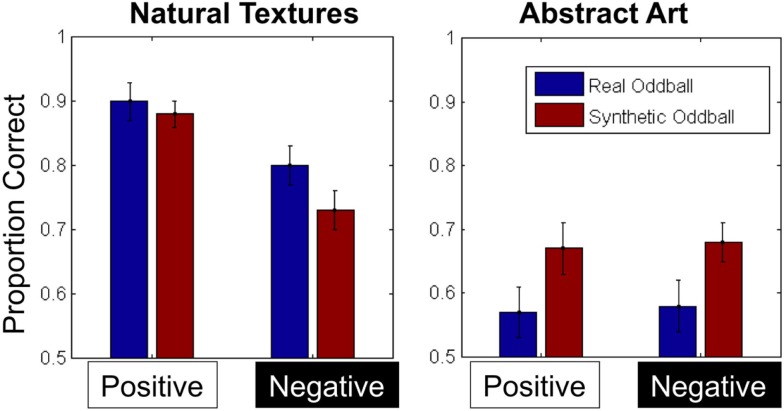
**Average proportion correct for all conditions in Experiment 1**. Error bars represent ±1 standard error of the mean.

To analyze our accuracy data, we calculated each participants’ proportion correct in each condition (Figure 3) and analyzed the results with a 2 × 2 × 2 repeated-measures ANOVA with texture category (fruit/vegetable vs. abstract art), contrast polarity (positive vs. negative), and oddball type (real vs. synthetic) as within-subject factors. We note that given our data is binomial (correct/incorrect) it is more proper to use probit or logit analysis instead of the linear model that is typically employed in similar studies, and so in both Experiments 1 and 2, we also carried out a logit analysis of our data. In each case, the results of obtained with the logit function were highly consistent with the results obtained from the linear model, and here we report the latter on the grounds that most readers are likely to be more familiar with this than the alternative.

The ANOVA revealed main effects of category [*F*(1, 12) = 58.1, *p* < 0.001, η^2^ = 0.83] and contrast [*F*(1, 12) = 19.55, *p* = 0.001, η^2^ = 0.62], indicating that discrimination performance was less accurate for abstract textures and contrast-negated textures. The main effect of contrast negation was qualified by an interaction between texture category and contrast, however [*F*(1, 12) = 22.23, *p* = 0.001, η^2^ = 0.65], such that the impact of contrast negation on performance was significantly reduced in the abstract art conditions. Finally, we also observed an interaction between texture category and oddball type [*F*(1, 12) = 5.80, *p* = 0.033, η^2^ = 0.33], such that synthetic oddballs were easier to detect in the abstract art conditions, but real oddballs were easier to detect in the fruit/vegetable conditions. No other main effects or interactions were significant.

### Discussion

The results of Experiment 1 reveal several interesting features of natural texture processing. First, we find that contrast negation of the entire stimulus array does affect the ability to discriminate real from synthetic textures, but not for textures of abstract artwork. This suggests that contrast negation removes information from the stimulus array that observers can use for discriminating between real and synthetic textures. Participants must use fairly complex statistics to identify the target in this task at all, since our real and synthetic textures have well-matched intensity histograms, orientation histograms, and joint histograms that describe several types of wavelet correlations. One typical proxy for “higher-order” statistics in natural textures, the phase spectrum (Emrith et al., 2010), also does not offer a good account of the effect of negation in our task. Following contrast negation, the phase of each image is rotated 180°, but since this is true for all the images in the array, numerical comparisons between the target and the distractors do not change. The information lost must thus be some other property of the textures used here, and the dependency of the negation effect on texture category suggests that this information is irrelevant or unavailable for comparisons between real and synthetic abstract artwork.

Second, we also observed an interaction between the effect of oddball type and texture category. We suggest that these results indicate that homogenization of texture appearance following synthesis is more evident for fruit/vegetable textures than abstract art. We interpret this detection asymmetry as evidence for homogenization based on Rosenholtz’s (2001) argument that many visual search asymmetries can be interpreted in terms of systematic differences in appearance variation in the two categories under consideration. An alternative interpretation of our data would be to hypothesize that the synthesis process removes some critical image feature that is present in the target texture, and which can be detected pre-attentively by the human visual system. We cannot explicitly rule out this possibility, but there are two primary reasons why we think it is a less parsimonious account of our results. First, as Rosenholtz (2001) discusses, hypothesizing new pre-attentive features in general leads to an unwieldy theory of what the visual system is computing – the description of the visual system is more of a laundry list of potential features than it is a theory based on consistent computational principles. Second, positing a true feature-based asymmetry depends on careful consideration of the experimental design in an appropriate feature space to ensure that there are not built-in asymmetries present. Given that we do not as yet know the right feature space for evaluating natural textures like those used here, we suggest that we cannot legitimately claim that our asymmetry is the result of a pre-attentively detected feature. However, regardless of which interpretation one favors, both this effect of the oddball and the overall effect of negation could be the result of material properties being difficult to recover from transformed texture images. The abstract textures we used in this task lacked qualities like glossiness (Anderson and Kim, 2009), translucency (Fleming and Bulthoff, 2005), or roughness, all of which depend in part on high-order statistics that are not generally preserved in synthetic or negated textures. Removing material properties could homogenize texture appearance as we hypothesize, or material properties could be an example of an image feature that is detected pre-attentively.

We continued in Experiment 2 by asking participants to perform a similar oddball detection task as described in Experiment 1, but with a design that complements the manipulations we implemented in our first task. In this case, we asked participants to detect an oddball texture that was defined by contrast polarity, subject to a transformation of the entire stimulus array via texture synthesis. This second task allowed us to determine if contrast negation and texture synthesis disrupt texture discrimination in a similar manner, or if these transformations differentially disrupt natural texture appearance.

## Experiment 2

In Experiment 2, we examined how participants’ ability to distinguish between natural and contrast-negated textures was impacted by the removal of higher-order statistics via texture synthesis. As in Experiment 1, we used a 4AFC oddball detection paradigm to further examine how the type of target and texture category affected performance in this task.

### Methods

#### Subjects

We recruited 11 participants (four female) to take part in Experiment 2. Participants were between the ages of 18–22 years old and self-reported normal or corrected-to-normal vision. None of the participants who volunteered for Experiment 2 had taken part in Experiment 1.

#### Stimuli

We used the same stimulus set described in Experiment 1 for this task.

#### Procedure

**Figure 4:**
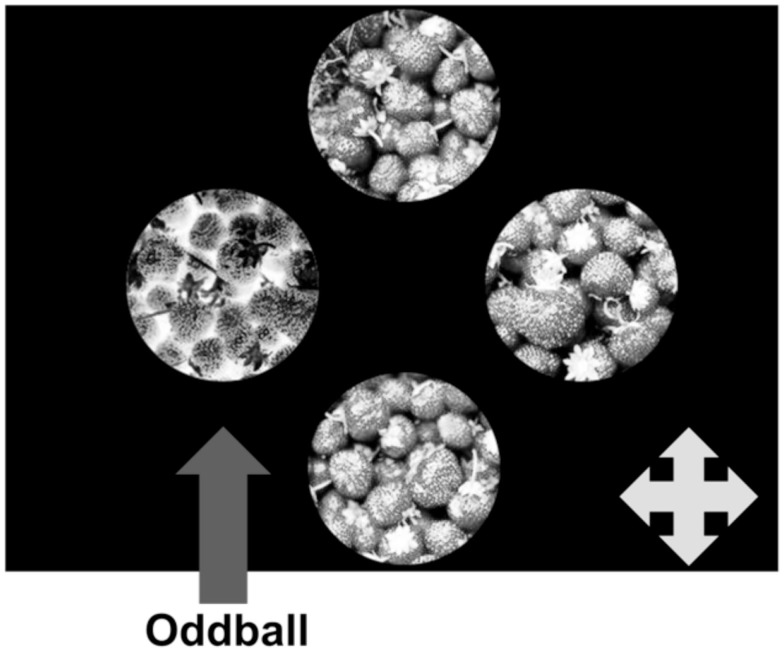
**Schematic representation of a single trial in Experiment 2**. Like Experiment 1, we used four unique texture patches on each trial. In this task, however, the oddball is of opposite contrast polarity than the distractors. A contrast-negated oddball is shown here.

We used the same paradigm in Experiment 2 as described above for Experiment 1. Participants were asked to select the oddball texture patch from an array of four unique items. The critical difference between this task and Experiment 1 is that the oddball in this task is defined by contrast polarity (positive or negative). Thus, the target texture can either be the only contrast-negated image among positive distractors or the converse (Figure 4). As in Experiment 1, we manipulated texture category in separate blocks that were alternated for presentation order across participants, and varied the type of oddball randomly within these blocks. Further, to examine the extent to which discriminating positive and negative texture patches depends upon a rich set of natural image statistics, we presented participants with arrays composed entirely of real textures as well as arrays comprised only of synthetic textures. The real/synthetic appearance of the entire array was randomized across trials within each category block.

All stimulus and display parameters were identical to those described in Experiment 1. Again, all experimental procedures and recruitment methods were approved by the North Dakota State University IRB.

#### Results

**Figure 5:**
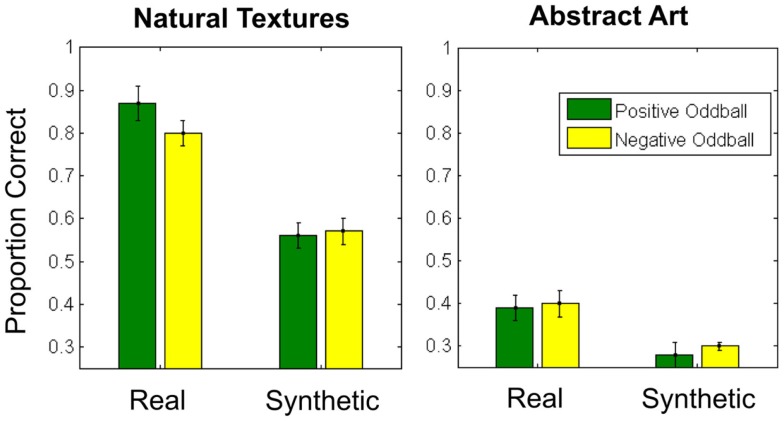
**Average proportion correct for all conditions in Experiment 2**. Error bars represent ±1 standard error of the mean.

As in Experiment 1, we determined participants’ accuracy in each condition (Figure 5) and submitted these values to a 2 × 2 × 2 repeated-measures ANOVA, with texture category (fruits/vegetables vs. abstract art), texture appearance (real vs. synthetic), and oddball type (positive vs. negative) as within-subjects factors.

The ANOVA revealed main effects of category [*F*(1, 10) = 385.2, *p* < 0.001, η^2^ = 0.98] and texture appearance [*F*(1, 10) = 120.7, *p* < 0.001, η^2^ = 0.92], indicating poorer performance with abstract art textures and synthesized texture patches. We also observed an interaction between category and texture appearance [*F*(1, 10) = 21.8, *p* = 0.001, η^2^ = 0.69], such that the impact of texture synthesis on performance appears to be reduced (but not absent) in the abstract art category relative to the fruit/vegetable textures. No other main effects or interactions reached significance. As in Experiment 1, we repeated this analysis with a logit link function, which yielded results consistent with this analysis.

### Discussion

The key results of Experiment 2 are twofold: first, texture synthesis does remove information observers use to discriminate between positive- and negative-contrast texture patches. While we did observe an interaction with texture category, unlike Experiment 1, this is likely due to a floor effect for detecting the oddball in the abstract artwork condition. The synthesized arrays are harder for participants, but performance with the original textures was already quite poor, leading to an interaction based on the size of an effect in the same direction as we obtained with fruit/vegetable textures. Second, we found no evidence of an oddball effect in this task – accuracy did not differ as a function of whether the oddball was of positive or negative polarity. Unlike texture synthesis, contrast negation does not appear to induce any changes in appearance that alter the homogeneity of texture patches. Contrast negation and texture synthesis thus both remove information that observers can use to discriminate between natural and unnatural textures, but the losses incurred by these transformations differ in their scope, and also differ in terms of their impact on texture homogeneity.

## General Discussion

The results of Experiment 1 and Experiment 2 extend Balas’ (2006) initial study of critical features for natural texture perception in several ways. In Experiment 1, we have demonstrated that discriminating natural from synthetic textures (a task that ostensibly requires observers to detect differences in summary statistics that are not explicitly matched by the Portilla–Simoncelli model) is affected by contrast negation in a category-dependent way. Contrast negation of the entire stimulus array preserves the amplitude spectrum of the constituent texture patches and also does not impact observers’ ability to use diagnostic information in the phase spectrum for discrimination. The rotation of the entire phase spectrum through 180° does not, for example, disrupt either local phase congruence (Morrone and Burr, 1988) or the phase computations explored by Phillips and Todd (2010) as a means by which macroscopic structures can be characterized. The imposition of contrast negation must therefore disrupt different perceptually relevant features that observers use to distinguish between natural and synthetic textures. This may include material properties (Motoyoshi et al., 2007) used either on their own or as a proxy for local shape computations (Fleming et al., 2004). In Experiment 2, we demonstrated that discriminating natural textures on the basis of contrast polarity was similarly impaired (though with a less clear category-dependence) by the removal of higher-order statistics via texture synthesis. Therefore, observers must be using information beyond what is contained in the joint wavelet statistics used by the Portilla–Simoncelli model to distinguish between positive and negative contrast patches from the same parent texture.

One simple conclusion that follows from our results is that observers’ make use of a rich and complex set of summary statistics for the purposes of texture discrimination. This is commensurate with a number of recent findings. Anderson and Kim (2009) noted that though pixel skewness correlates with perceived glossiness in some circumstances, interactions between local surface geometry and local image statistics are also critical for material perception. Similarly, the “bumpiness” and glossiness of textured surfaces are measured conjointly (Ho et al., 2008). In both cases, texture synthesis (which can disrupt local conjunctions that support the recovery of surface geometry) and contrast negation (which can disrupt perceived glossiness and translucency) would be expected to disrupt material perception. The recovery of shape from texture is also known to be disrupted following synthesis of the original texture (Fleming et al., 2003), and the disruption of specularities by contrast negation may also disrupt shape computations that are based on texture appearance (Fleming et al., 2004). These transformations thus disrupt multiple mid- to high-level texture properties of natural textures, which we would expect to be relevant in our fruit/vegetable condition, but not necessarily in our abstract artwork condition.

Our data also imply that the visual system is tuned to natural texture appearance. We have found that specific deviations from natural texture statistics across the entire stimulus array lead to an impaired ability to discriminate texture patterns – this is the case when we consider abstract art textures as examples of unnatural textures, and also applies to contrast-reversed and synthetic textures. In each of these three cases, observers do more poorly when asked to discriminate between texture patches that do not match the typical appearance of natural textures. This result differs from reports that natural image statistics impair the detection of contrast boundaries (Arsenault et al., 2011) and distortions of local spatial structure (Bex, 2010). Specifically, we find that ecologically relevant texture appearance is facilitative, both when we consider the performance cost incurred by asking participants to discriminate between abstract artwork patterns relative to more ecologically valid textures and also when we consider the effects of our two manipulations of texture appearance. However, all three of these studies (the current result; Bex, 2010; and Arsenault et al., 2011) demonstrate that the visual system is in some sense tuned to the statistics of the natural world – the difference is how that tuning impacts performance.

Finally, the differential effect of oddball type in Experiments 1 and 2 suggests that contrast negation and texture synthesis do fundamentally different things to natural texture appearance. Specifically, contrast negation does not alter the perceived homogeneity of texture patches, while texture synthesis does so in a category-dependent manner (which may reflect ecological tuning of the visual system to a subset of texture categories). This distinction between how negation and texture synthesis differentially disrupt texture appearance may be related to Motoyoshi and Kingdom’s (2007) proposal that there are two streams of second-order processing: one stream that is sensitive to polarity (but not orientation), and another that is sensitive to orientation (but not polarity). Target detection in Experiments 1 and 2 may be primarily supported by orientation-sensitive and polarity-sensitive mechanisms, respectively, but it is unclear how the different properties of these streams would lead to the observed asymmetries of oddball type observed in Experiment 1. More broadly, contrast negation and texture synthesis (as operationalized here) may differ in the extent to which they disrupt the “things” and the “stuff” in texture images. Intuitively, texture synthesis may disrupt both qualities of a texture image while contrast negation may primarily disrupt the “stuff” in a texture, since the preservation of edge distributions following contrast negation may preserve observers’ ability to segment discrete objects. This is an appealing idea, and examining the differential properties of textures made up of objects and textures that lack segmentable structures could shed light on this issue. We note however, that “things” and “stuff” are likely to be disrupted by both transformations to some degree, especially since contrast negation is known to disrupt shape recovery in some circumstances (Fleming et al., 2004), which may subsequently lead to difficulties inferring the “things” that are in an image as well as the material properties.

The current results suggest several interesting questions for further research. First, we have used just one parametric texture synthesis model as the basis for measuring the extent to which higher-order statistics impact the discrimination of natural textures from unnatural textures. Selective lesioning of the model (Balas, 2006) or augmentation of its vocabulary could potentially inform us as to which statistics observers use in both of our tasks. Manipulating the parameters of the P-S model could also reveal important aspects of how natural texture discrimination is implemented – Freeman and Simoncelli (2011), for example, have created what they call texture “metamers” by varying the scale parameter across the periphery. By varying this parameter (and others) we could potentially examine how sensitive our results are to the amount of local integration used to estimate appearance. Second, some non-parametric texture synthesis techniques that use texture-quilting as a method for synthesizing textures (Efros and Freeman, 2001) guarantee complete preservation of texture appearance within a local neighborhood, but may introduce errors at larger scales subsequent to the assemblage of texture patches into a larger pattern. Varying the size of the constituent tiles in such a synthesized texture may thus be a useful means of examining the size of the spatial neighborhood observers use to compare natural textures to transformed ones, and how computations at different spatial scales are disrupted by the manipulations we have employed here. Attempts to adapt non-parametric techniques like pixel-growing (Efros and Leung, 1999) and quilting may help us investigate the spatial extent of texture processing even if these models are not so directly analogous to the human visual system. Finally, we have made a rather crude comparison here between two texture categories: images of food and images of abstract artwork. For our purposes, these categories were useful insofar as they differed substantially in terms of observers’ experience with them and the range of material properties, etc., that were present in each category. Our choice of stimuli raises the obvious question of generality, however – are our effects specific to fruit and vegetables? What differences between these categories drove the category interactions we observed? At a category level or an item level, considering the impact of specific image properties (e.g., edge density, similar to Arsenault et al., 2011) may help clarify the underlying mechanisms supporting these effects. Likewise, systematically varying mid- and high-level image properties across a wider range of categories (e.g., glossy and matte textures, dense vs. sparse textures) may yield further insight into what statistics observers make use of in natural texture discrimination tasks.

## Conflict of Interest Statement

The author declares that the research was conducted in the absence of any commercial or financial relationships that could be construed as a potential conflict of interest.

## References

[B1] Adelson E. H. Bergen J. R. (1991). “The plenoptic function and the elements of early vision,” in Computational Models of Visual Processing, eds Landy M. S. Movshon J. A. (Cambridge, MA: MIT Press), 3–20

[B2] Anderson B. L. Kim J. (2009). Image statistics do not explain the perception of gloss and lightness. J. Vis. 9, 1–17 10.1167/9.13.1 20053073

[B3] Arsenault E. Yoonessi A. Baker C. (2011). Higher order texture statistics impair contrast boundary segmentation. J. Vis. 11, 14 10.1167/11.7.14 21933932

[B4] Balas B. (2006). Texture synthesis and perception: using computational models to study texture representations in the human visual system. Vision Res. 46, 299–309 10.1016/j.visres.2005.04.013 15964047

[B5] Balas B. (2008). Attentive texture similarity as a categorization task: comparing texture synthesis models. Pattern Recognit. 41, 972–982 10.1016/j.patcog.2007.08.007 20890384 PMC2947373

[B6] Balas B. Nakano L. Rosenholtz R. (2009). A summary-statistic representation in peripheral vision explains visual crowding. J. Vis. 9, 13 10.1167/9.2.13 20053104 PMC2923917

[B7] Beck J. (1983). Textural segmentation, second-order statistics, and textural elements. Biol. Cybern. 48, 125–130 10.1007/BF00344396 6626590

[B8] Bex P. J. (2010). (In) Sensitivity to spatial distortion in natural scenes. J. Vis. 10, 23 10.1167/10.14.23 20462324 PMC2924673

[B9] Brainard D. H. (1997). The Psychophysics Toolbox. Spat. Vis. 10, 433–436 10.1163/156856897X00357 9176952

[B10] Chubb C. Econopouly J. Landy M. S. (1994). Histogram contrast analysis and the visual segregation of IID textures. J. Opt. Soc. Am. A Opt. Image Sci. Vis. 11, 2350–2374 10.1364/JOSAA.11.002350 7931761

[B11] Chubb C. Landy M. S. Econopouly J. (2004). A visual mechanism tuned to black. Vision Res. 44, 3223–3232 10.1016/j.visres.2004.07.019 15482808

[B12] Efros A. A. Freeman W. T. (2001). Image Quilting for Texture Synthesis and Transfer, SIGGRAPH, Los Angeles, CA

[B13] Efros A. A. Leung T. K. (1999). “Texture synthesis by nonparametric sampling,” in Proceedings of the International Conference on Computer Vision, Corfu, 1033–1038

[B14] Emrith K. Chantler M. J. Green P. R. Maloney L. T. Clarke A. D. F. (2010). Measuring perceived differences in surface texture due to changes in higher order statistics. J. Opt. Soc. Am. A Opt. Image Sci. Vis. 27, 1232–1244 20448792 10.1364/JOSAA.27.001232

[B15] Fleming R. Bulthoff H. H. (2005). Low-level image cues in the perception of translucent materials. ACM Trans. Appl. Percept. 2, 346–382 10.1145/1077399.1077409

[B16] Fleming R. W. Dror R. O. Adelson E. H. (2003). Real-world illumination and the perception of surface reflectance properties. J. Vis. 3, 347–368 10.1167/3.5.3 12875632

[B17] Fleming R. W. Holtmann-Rice D. Bulthoff H. H. (2011). Estimation of 3D shape from image orientations. Proc. Natl. Acad. Sci. U.S.A. 108, 20438–20443 10.1073/pnas.1114619109 22147916 PMC3251077

[B18] Fleming R. W. Torralba A. Adelson E. H. (2004). Specular reflections and the perception of shape. J. Vis. 4, 798–820 10.1167/4.8.798 15493971

[B19] Freeman J. Simoncelli E. P. (2011). Metamers of the ventral stream. Nat. Neurosci. 14, 1195–1201 10.1038/nn.2889 21841776 PMC3164938

[B20] Heeger D. J. Bergen J. R. (1995). “Pyramid-based texture analysis/synthesis,” in Proceedings of the 22nd Annual Conference on Computer Graphics & Interactive Techniques, Los Angeles, Vol. 30, 229–238

[B21] Ho Y.-X. Landy M. S. Maloney L. T. (2008). Conjoint measurement of gloss and surface texture. Psychol. Sci. 19, 196–204 10.1111/j.1467-9280.2008.02067.x 18271869 PMC2679902

[B22] Julesz B. (1981). A theory of preattentive texture discrimination based on the first order statistics of textons. Biol. Cybern. 41, 131–138 10.1007/BF00335367 7248342

[B23] Julesz B. Gilbert E. N. Victor J. D. (1978). Visual discrimination of textures with identical third-order statistics. Biol. Cybern. 31, 137–140 10.1007/BF00336998 728493

[B25] Lettvin J. Y. (1976). On seeing sidelong. Science 16, 10–20

[B26] Li A. Zaidi Q. (2000). Perception of three-dimensional shape from texture is based on patterns of oriented energy. Vision Res. 40, 217–242 10.1016/S0042-6989(00)00212-1 10793898

[B27] Li A. Zaidi Q. (2001). Veridicality of three-dimensional shape perception predicted from amplitude spectra of natural textures. J. Opt. Soc. Am. A Opt. Image Sci. Vis. 18, 2430–2447 10.1364/JOSAA.18.002969 11583260

[B28] Malik J. Belongie S. Leung T. Shi J. (2001). Contour and texture analysis for image segmentation. Int. J. Comput. Vis. 43, 7–27 10.1023/A:1011122819729

[B29] Malik J. Perona P. (1990). Preattentive texture discrimination with early vision mechanism. J. Opt. Soc. Am. A Opt. Image Sci. Vis. 5, 923–932 10.1364/JOSAA.7.000923 2338600

[B30] Morrone M. C. Burr D. C. (1988). Feature detection in human vision: a phase-dependent energy model. Proc. R. Soc. Lond. B Biol. Sci. 235, 221–245 10.1098/rspb.1988.0073 2907382

[B31] Motoyoshi I. Kingdom F. A. A. (2007). Differential roles of contrast polarity reveal two streams of second-order visual processing. Vision Res. 47, 2047–2054 10.1016/j.visres.2007.03.015 17555787

[B32] Motoyoshi I. Nishida S. Sharan L. Adelson E. H. (2007). Image statistics and the perception of surface qualities. Nature 447, 206–209 10.1038/nature05724 17443193

[B33] Padilla S. Drbohlav O. Green P. R. Spence A. Chantler M. J. (2008). Perceived roughness of 1/f! Noise surfaces. Vision Res. 48, 1791–1797 10.1016/j.visres.2008.05.015 18603278

[B34] Pelli D. G. (1997). The VideoToolbox software for visual psychophysics: transforming numbers into movies. Spat. Vis. 10, 437–442 10.1163/156856897X00366 9176953

[B35] Phillips F. Todd J. T. (2010). Texture discrimination based on global feature alignments. J. Vis. 10, 6 10.1167/10.10.6 20884555

[B36] Portilla J. Simoncelli E. (2000). A parametric texture model based on joint statistics of complex wavelet coefficients. Int. J. Comput. Vis. 40, 49–71 10.1023/A:1026553619983

[B37] Rosenholtz R. (2000). “Significantly different textures: a computational model of pre-attentive texture segmentation,” in Proceedings of the European Conference on Computer Vision, LNCS 1843, ed. Vernon J. (Dublin: Springer Verlag), 197–211

[B38] Rosenholtz R. (2001). Search asymmetries? What search asymmetries? Percept. Psychophys. 63, 476–489 10.3758/BF03194414 11414135

[B39] Rosenholtz R. (2011). What your visual system sees where you are not looking, Proceedings of SPIE, Human Vision and Electronic Imaging, San Francisco

[B40] Rosenholtz R. Huang J. Ehinger K. A. (2012a). Rethinking the role of top-down attention in vision: effects attributable to a lossy representation in peripheral vision. Front. Psychol. 3:13 10.3389/fpsyg.2012.00013 22347200 PMC3272623

[B41] Rosenholtz R. Huang J. Raj A. Balas B. J. Ilie L. (2012b). A summary statistic representation in peripheral vision explains visual search. J. Vis. 12, 1–17 10.1167/12.3.1 PMC4032502 22523401

[B42] Sharan L. Li Y. Motoyoshi I. Nishida S. Adelson E. H. (2008). Image statistics for surface reflectance perception. J. Opt. Soc. Am. 25, 846–864 10.1364/JOSAA.25.000846 18382484

[B43] Taylor R. P. Spehar B. Van Donkelaar P. Hagerhall C. M. (2011). Perceptual and physiological responses to Jackson Pollack’s Fractals. Front. Hum. Neurosci. 5:60 10.3389/fnhum.2011.00060 21734876 PMC3124832

[B44] Tkacik G. Prentice J. S. Victor J. D. Balasubramanian V. (2010). Local statistics in natural scenes predict the saliency of synthetic textures. Proc. Natl. Acad. Sci. U.S.A. 107, 18149–18154 10.1073/pnas.1004906107 20923876 PMC2964243

[B45] Tyler C. W. (2004). Beyond fourth-order texture discrimination: generation of extreme-order and statistically balanced textures. Vision Res. 44, 187–2199 10.1016/j.visres.2004.03.029 15183686

